# Update on the ESCEO recommendation for the conduct of clinical trials for drugs aiming at the treatment of sarcopenia in older adults

**DOI:** 10.1007/s40520-020-01663-4

**Published:** 2020-07-31

**Authors:** Jean-Yves Reginster, Charlotte Beaudart, Nasser Al-Daghri, Bernard Avouac, Jürgen Bauer, Nathalie Bere, Olivier Bruyère, Francesca Cerreta, Matteo Cesari, Mario Miguel Rosa, Cyrus Cooper, Alfonso J. Cruz Jentoft, Elaine Dennison, Anton Geerinck, Evelien Gielen, Francesco Landi, Andrea Laslop, Stefania Maggi, María Concepción Prieto Yerro, René Rizzoli, Hildrun Sundseth, Cornel Sieber, Andrea Trombetti, Bruno Vellas, Nicola Veronese, Marjolein Visser, Mila Vlaskovska, Roger A. Fielding

**Affiliations:** 1 WHO Collaborating Center for Public Health Aspects of Musculo-Skeletal Health and Ageing, Division of Public Health, Epidemiology and Health Economics, University of Liège, Avenue Hippocrate 13, CHU B23, 4000 Liege, Belgium; 2 Chair for Biomarkers of Chronic Diseases, Biochemistry Department, College of Science, King Saud University, Riyadh, 11451 Saudi Arabia; 3 Department of Rheumatology, CHU Henri Mondor, Creteil, France; 4 Center for Geriatric Medicine and Network Aging Research, Heidelberg University, Heidelberg, Germany; 5 European Medicines Agency, Amsterdam, The Netherlands; 6 Department of Clinical Sciences and Community Health, University of Milan, Milan, Italy; 7 Geriatric Unit, Fondazione IRCCS Ca’ Granda Ospedale Maggiore Policlinico, Milan, Italy; 8 Departamento de Neurociências/Laboratório de Farmacologia Clínica E Terapêutica, Faculdade de Medicina da Universidade de Lisboa, Lisbon, Portugal; 9 MRC Lifecourse Epidemiology Unit, University of Southampton, Southampton, UK; 10 Hospital Universitario Ramón Y Cajal (IRYCIS), Madrid, Spain; 11 Division of Geriatrics, UZ Leuven, Leuven, Belgium; 12 Department of Geriatrics, Neurosciences and Orthopedics, Catholic University of the Sacred Heart Rome, Milan, Italy; 13 Scientific Office, Federal Office for Safety in Health Care, Vienna, Austria; 14 CNR Aging Branch-IN, Padua, Italy; 15 Agencia Española de Medicamentos Y Productos Sanitarios, Madrid, Spain; 16 Division of Bone Diseases, Geneva University Hospitals and Faculty of Medicine, Geneva, Switzerland; 17 European Institute of Women’s Health, Dublin, Ireland; 18 Institute for Biomedicine of Aging Friedrich-Alexander University Erlangen-Nürnberg, Erlangen, Germany; 19 Department of Internal and Geriatrics Medicine, Gerontopole, CHU de Toulouse, UMR 1027 INSERM, University Toulouse III, Toulouse, France; 20 Department of Internal Medicine, Geriatrics Section, University of Palermo, Palermo, Italy; 21 Department of Health Sciences, Vrije Universiteit Amsterdam, Amsterdam, The Netherlands; 22 Medical Faculty, Department of Pharmacology and Toxicology, Medical University Sofia, Sofia, Bulgaria; 23 Nutrition, Exercise Physiology and Sarcopenia Laboratory, Jean Mayer USDA Human Nutrition Research Center On Aging, Tufts University, Boston, USA; 24 Gerontology and Geriatrics, Department of Public Health and Primary Care, KU Leuven, Leuven, Belgium; 25 Kantonsspital Winterthur, Winterthur, Switzerland

**Keywords:** Clinical trial, Sarcopenia, Guidelines, Recommendations, Drug registration, Treatment

## Abstract

**Background:**

In 2016, an expert working group was convened under the auspices of the European Society for Clinical and Economic Aspects of Osteoporosis and Osteoarthritis (ESCEO) and formulated consensus recommendations for the conduct of clinical trials for drugs to prevent or treat sarcopenia.

**Aims:**

The objective of the current paper is to provide a 2020 update of the previous recommendations in accordance with the evidence that has become available since our original recommendations.

**Methods:**

This paper is based on literature reviews performed by members of the ESCEO working group and followed up with face to face meetings organized for the whole group to make amendments and discuss further recommendations.

**Results:**

The randomized placebo-controlled double-blind parallel-arm drug clinical trials should be the design of choice for both phase II and III trials. Treatment and follow-up should run at least 6 months for phase II and 12 months for phase III trials. Overall physical activity, nutrition, co-prescriptions and comorbidity should be recorded. Participants in these trials should be at least 70-years-old and present with a combination of low muscle strength and low physical performance. Severely malnourished individuals, as well as bedridden patients, patients with extremely limited mobility or individuals with physical limitations clearly attributable to the direct effect of a specific disease, should be excluded. Multiple outcomes are proposed for phase II trials, including, as example, physical performance, muscle strength and mass, muscle metabolism and muscle-bone interaction. For phase III trials, we recommend a co-primary endpoint of a measure of functional performance and a Patient Reported Outcome Measure.

**Conclusion:**

The working group has formulated consensus recommendations on specific aspects of trial design, and in doing so hopes to contribute to an improvement of the methodological robustness and comparability of clinical trials. Standardization of designs and outcomes would advance the field by allowing better comparison across studies, including performing individual patient-data meta-analyses, and different pro-myogenic therapies.

## Introduction

In 2016, a panel of experts from different disciplines reviewed and discussed the evidence available at the time and formulated consensus recommendations for the conduct of clinical trials for drugs to prevent or treat sarcopenia, under the auspices of the European Society for Clinical and Economic Aspects of Osteoporosis and Osteoarthritis (ESCEO) [1]. In the intervening years, important strides have been made in the field of sarcopenia, notably with regard to diagnostic criteria and its epidemiological characteristics [2]. The range of pharmacological therapies under investigation has expanded, although the earlier flurry of pharmacological therapies has subsided. However, among the pharmacological interventions that have been investigated few have shown positive results [3], which has led researchers to ask whether we missed a drug because we did not design the right trial for it. Both the absence of shared procedures and outcomes between clinical trials, differences in sample selection, as well as the uncertainty about the required duration of follow-up may have impacted results of previous clinical trials in sarcopenia. Furthermore, the current literature is confusing, with studies using diverse inclusion/exclusion criteria, treatment durations, concomitant therapies (e.g., exercise, nutrition interventions), and study outcome measures, complicating comparison of results [4].

In the 2016 recommendations, the working group members highlighted that a crucial next step in the evolution of sarcopenia research would be to find an agreement on an operational definition for sarcopenia, with accepted thresholds for abnormal muscle mass and muscle function [1]. In recent years, consensus has consolidated upon three consensus definitions, one European [European Working Group on Sarcopenia in Older People (EWGSOP)], one Asian [Asian Working Group on Sarcopenia (AWGS)] and one American [Sarcopenia Definitions and Outcome Consortium (SDOC)] [2, 5, 6]. The EWGSOP published its revised consensus statement in 2019, wherein they refined their previous diagnostic criteria and proposed an algorithm for case-finding. This group defined age-related sarcopenia, as “a progressive and generalized skeletal muscle disorder that is associated with increased likelihood of adverse outcomes including falls, fractures, physical disability and mortality” [2]. The diagnosis of sarcopenia in the EWGSOP2 and AWGS definitions established through a combination of low muscle mass and low muscle function (strength and/or physical performance), but cut-off values and proposed tests and instruments differ between them [2, 5]. The recent definition from the SDOC [6] takes a different approach to the diagnosis of sarcopenia. An international expert panel, guided by findings from 18 studies and literature reviews, voted on 13 position statements on grip strength, lean mass measured by Dual Energy X-ray absorptiometry (DXA), gait speed, and two summary statements. The panel strongly agreed that both low grip strength and low usual gait speed should be included in the definition of sarcopenia, and that these two indicators independently predict adverse outcomes in community-dwelling older people [6]. Although most efforts have been focused on age-related sarcopenia, also called primary sarcopenia, there is increasing awareness of the different clinical situations in which sarcopenia can present in an acute form, such as bedbound patients in hospital settings. Secondary sarcopenia occurs when other causal factors (in combination with or besides ageing) are at the source of the observed muscle wasting. Among these factors are comorbidities such as organ failure, inflammatory diseases, cancer and endocrine diseases; malnutrition, possibly due to gastrointestinal disorders, anorexia or psychosocial disorders; and inactivity due to a multitude of reasons [7].

Since 2016, age-related sarcopenia has been recognized as a muscle disease through an ICD-10-Clinical Modification code (M62.84) [8]. This code allows for sarcopenia to be recognized as a reportable condition by the US Food and Drug Administration (FDA) and the European Medicines Agency (EMA), and provides an additional argument for increased investment by industry and non-profit organizations into pharmacological interventions designed to improve sarcopenia-related outcomes [9].

In the past few years, a number of pharmacological interventions have been studied in clinical trials to understand whether they might prove to be effective in reducing or reversing the loss of muscle mass, muscle strength and physical function that characterizes sarcopenia [10, 11]. Although a pharmacological agent has not been authorized yet, several pharmacologic agents have been reported to be under investigation. Rooks and Roubenoff provided an overview of the pharmacological approaches as of the end of 2018, detailing the different mechanisms of action such as the selective androgen receptor modulators (SARMS) and drugs that target the myostatin activin pathway. They list selective androgen receptor modulators, activin receptor agonists, myostatin or activin inhibitors fast skeletal muscle troponin activators and the Mas-receptor in the renin-angiotensin system [12]. In April 2020, a search on clinicaltrials.gov database for all clinical trials that listed sarcopenia as one target condition, yielded 461 study protocols. After manual selection, 44 interventional trials studying pharmacological interventions were withheld, the details of which can be found in appendix 1.

A second expert working group composed of clinicians, researchers, representatives of the regulatory bodies and a patient advocate was convened in February of 2020 under the auspices of ESCEO. The present paper provides an update of the previous recommendations in accordance with the evidence that has become available in the meantime, to stimulate the debate about the design of phase II and phase III clinical trials in sarcopenia taking into account knowledge obtained in the last years.

## Methods

In light of the evolution in pharmacological treatment options since 2016, and the need to link the current academic and regulatory perspectives, a second expert working group was convened in February of 2020 under the direction of ESCEO. This group assembled clinicians and researchers, as well as representatives of the regulatory bodies and a patient advocate. Certain members of the core writing group were asked to review the literature and/or to present the current state of the art on the following topics: (1) current status of sarcopenia drug development (FC); (2) did clinical trials in sarcopenia fail because of inappropriate design? (RF); (3) the impact of the EWGSOP2 sarcopenia definition on the selection of target populations for phase II and III trials (AC-J); (4) the identification of secondary endpoints (MC and FL); (5) patient-reported outcome measures in sarcopenia (CB); and (6) specific design aspects for clinical trials in sarcopenia (JB). The core writing group participated in presentations and discussion rounds, and created the preliminary version of these recommendations. A larger consensus group was solicited to provide written comments via email on the preliminary recommendations, which ultimately led to the consensus described in this article.

## Results

### Target population for pharmacological treatment RCTs in sarcopenia

#### Inclusion criteria

Potential target populations that should be included in pharmaceutical trials in sarcopenia have already been discussed in past publications [1, 13–16]. Based on these previous publications, as well as on the experience acquired in the field of sarcopenia since and ESCEO expert’s opinion, we advocate to recruit patients, both for phase II or phase III trials, fulfilling the following criteria:

- Sex: both
- Age: 70 years and older with no upper age limit. Even if traditionally, 65 has been taken as the entry point into old age, working patterns are shifting and, in Western countries, it is often 70 years that is now considered as a threshold for old age. Moreover, the purpose of a clinical trial is obviously to obtain a benefit for the participants; to evaluate whether an intervention is beneficial, sarcopenia-related impairments must therefore be present to a relevant degree in the included population. Considering that the prevalence of sarcopenia has been shown to increase with age, the ESCEO working group recommends the inclusion of individuals with a minimal age limit of 70 years [17, 18].
- Physical abilities:
  o *Able to walk*, and preferably able to perform the 400-m walk test within 15 min and without sitting, leaning or the help of another person or walker. This inclusion criterion is important as many events results from deambulation (falls, fractures, pain) and may be of high relevance in the context of primary and secondary outcome measurements.
  o *Presenting a combination of a low grip strength* + *low physical performance*.
    We recommend identifying the target population through objective measures of muscle strength and physical performance. Participants could be enrolled in a trial if they present both a reduction of their muscle strength capacities and of their physical performance capacities [6, 16]. This “sarcopenia risk profile” could optimize the probability to detect a meaningful difference between intervention and control group.
    We acknowledge that muscle mass has been an important diagnostic criterion for sarcopenia. But, so far, low muscle mass has been shown to be less related to health-related outcomes, as compared with other sarcopenia criteria. Moreover, there are still important methodological issues with regard to the measurement of muscle mass. Therefore, interventions for sarcopenia should not be restricted to sarcopenic patients, thus not be limited to participants presenting with low muscle mass combined with low muscle strength. To enlarge inclusion criteria, it has been decided to leave out muscle mass as inclusion criterion (at least, to date), but we underline that this proposal only applies to inclusion criteria and not to outcome measurement.
    The validated cut-offs proposed for the criteria low grip strength and low physical performance are available in Table 1. Any of the proposed tools could be used without restriction, depending on the logistic capacities of the recruitment centre. The most appropriate procedure of administration, equipment, performance (i.e., feasibility, reliability and floor/ceiling effects) and well as the references range of each of these tools have already been described in a previous publication endorsed by ESCEO [19].

**Table 1:** Inclusion criteria related to muscle strength and physical performance

	Tool proposed	Cut-offs proposed
Low muscle strength	Hydraulic handheld isometric dynamometer (i.e., JAMAR or similar)	Maximal value from three attempts for each hands [20]: < 27 kg for men; < 16 kg for women [21]
	Chair stand test	> 15 s for five rises [22]
Low physical performance	SPBB test	≤ 8 points [23]
	Gait speed (4-m)	≤ 0.8 m/s [24]
	Timed up and go	≥ 20 s [25]

*SPPB* short physical performance battery

#### Exclusion criteria

- Nutritional status of all participants should be evaluated during the recruitment stage, and those who are severely malnourished should not be included in the trial. Malnutrition could be measured using one of the several available diagnostic tool; Mini-Nutritional assessment tool, European Society of Clinical Nutrition and Metabolism (ESPEN) criteria, Global Leadership Initiative of Malnutrition (GLIM) criteria, etc. [26–28], Indeed, malnutrition has been shown to influence muscle mass and is a strong predictor of sarcopenia. Moreover, malnourished participants have been shown to be at higher risk of mortality over a short-term period [29, 30].
- Patients with acute immobility (i.e., post hip fracture or post-acute hospital admission) should be excluded.
- Patients suffering from specific advanced pathologies such as, for example, terminal cancer, severe renal diseases [e.g., Estimated Glomerular Filtration Rate (eGFR) < 30], chronic obstructive pulmonary diseases (COPD) requiring oxygen, etc. should not be included in the clinical trial.
- Factors that may affect conduct of the trial (e.g., physical limitations should not be clearly attributable to the direct effect of a specific disease other than sarcopenia). In particular, patients with a physical limitation clearly attributable to the direct effect of a specific disease other than sarcopenia should be excluded (e.g., patients with a diagnosis of dementia or score < 24 on the Mini Mental State Examination (MMSE), patients with serious neurological, neuromuscular or orthopaedic conditions (e.g., Parkinson’s disease), patients with thymic disorders (e.g., anxious or depressive syndrome)).

Considering the number of different pathways linked to the sarcopenia and the large heterogeneity of the older adult population, exclusion criteria recommended could depend on the target (age-related primary sarcopenia only, secondary sarcopenia only, or both), on mechanism of action of the drug/agent/therapy, and on the clinical setting. Research teams should consider these specificities before finalizing specific exclusion criteria.

The exclusion criteria are used to build more robust samples, reduce the rate of dropouts and enhance the effect of the intervention. However, having stringent exclusion criteria might lead to the predominant inclusion of people with primary sarcopenia. If the study is targeting another distinct population of patients, exclusion criteria might need be reconsidered and modified.

## Primary and secondary outcomes for pharmacological treatment RCTs in sarcopenia

### Phase II studies

Phase II trials are designed with two important objectives: 1/obtaining a “proof of concept” for the new entities, 2/allowing a clear assessment of the effective dose-range to highlight the best dose to use in phase III trials within an economically reasonable time frame.

Different primary endpoints could be proposed in phase II studies. As a primary consideration, measures with a higher rate of change over time may reduce the follow-up duration and could therefore be chosen as a primary endpoint in phase II trials. However, since the objective of phase II clinical trials is to demonstrate the efficacy of the intervention, the study design might be more flexible since it is based on surrogates of the condition of interest. Table 2 displays all outcomes that we considered as applicable in phase II studies.

**Table 2:** List of outcomes that could be applicable in phase II studies (the final choice is the responsibility of the applicant)

Proposed endpoint	Tool to measure the endpoint
Improvement of physical performance	SPPB
	Gait speed
	400-m walk test
	TUG test
	Chair stand test
Improvement of muscle strength	Handgrip strength
	Knee extensor strength
Increase of muscle mass	CT-scan
	MRI
	DXA
	Urinary D-3 creatine
	Muscle biopsy
Improvement of muscle quality*	CT-scan
	MRI
	MRS
	Muscle biopsy
Muscle metabolism	Biomarkers of muscle metabolism
Muscle-bone interaction	Biomarkers of muscle-bone interaction (e.g., Myostatin, Activin A, amino terminal of type III procollagen peptide (P3NP), insulin-like growth factor-1 (IGF-1) [31, 32])

*For example, decrease in intramyocellular lipid accumulation and intermuscular adipose tissue, increase in muscle blood flow, change in fibre types and increase in skeletal muscle mitochondrial capacity

*MRS* magnetic resonance spectroscopy, *SPPB* short physical performance battery, *TUG* timed Up and Go, *CT-scan* computerized tomography scan, *MRI* magnetic resonance imaging

The choice of outcome to be used in phase II trial as primary endpoint—which is the responsibility of the applicant—should be done according to the hypothesized mode of action of the drug.

### Phase III studies

Phase III studies are pivotal for the applicant to demonstrate that the currently investigated new chemical entity is safe and effective for the management of sarcopenia.

#### Primary endpoint

This outcome should be clinically relevant, highly responsive to treatment effects and methodologically robust. The primary outcome in phase III studies is used to calculate the necessary sample size and to inform the randomization procedures.

We recommend, for all phase III clinical trials in sarcopenia, the use of co-primary endpoints, combining a measure of physical performance with a Patient Reported Outcome Measure (PROM). With this proposal, we ensure to capture first an objective endpoint, by recommending a physical performance measure, for which a surrogate value for hard clinical endpoints such as mortality, hospitalisation, fractures and falls is already well established [33]. Second, the use of a PROM will allow to capture more subjective but equally important aspects of patient-relevant efficacy since the patient perspective is now recognized as key parameters in the evaluation of health interventions [34, 35]. There is an increasing emphasis on patient-centred research and PROMs are increasingly recognized by government regulatory agencies (e.g., FDA, EMA, etc.), clinicians and patients as valuable tools to be used in clinical trials to ensure the impact of a clinical intervention is comprehensively assessed [36]. The way of how patients perceive the benefit derived from an intervention could be captured by the use of PROM. A common issue of co-primary endpoints appears when the two co-endpoints respond differently to the treatment [37, 38]. However, combining an objective primary endpoint such as physical performance with a subjective well-being endpoint such as quality of life reduces the risk for this scenario. Indeed, previous studies have already highlighted the linear relationship between the improvement of physical performance and health-related quality of life in older adults [39, 40]. This combination of physical performance and PRO is also likely to be relevant for Health Technology (reimbursement) assessment.

Physical performance is clinically relevant, considered as most important by patients themselves as compared with muscle strength and muscle mass, easy to implement in clinical settings and closely related to muscle health. Moreover, normative data and thresholds to define meaningful change are available for this outcome [41]. Among the different instruments available to measure physical performance, the 400-m walk test seems to best reflect autonomy [42]. Indeed, this distance is a common distance used to assess self-reported physical performance and believed to be required for independence with daily tasks. Moreover, inability to complete the 400-m walk test is highly associated with negative health outcomes (mortality, cardiovascular event, disability) and higher healthcare costs [42–44]. Finally, previous published data in intervention studies using the 400-m walk test as primary outcome are available for the calculation of sample size of any new clinical trial [17, 18]. We recommend measuring the incidence of inability to perform the 400-m walk test (also called mobility disability) as primary endpoint. Other tests, such as the SPBB test, deeply correlated with physical performance as measured by the 400-m walk test could also be proposed [45]. Moreover, SPPB is also linked to more distal clinical endpoints such as falls, fractures, nursing home admission and mortality [46, 47].

There are several reasons why muscle mass and muscle strength are not considered to be appropriate candidates for a primary endpoint. First, the different tools available to measure muscle mass and strength are very heterogeneous and no proper and valid gold standard has categorically been defined. Second, there is a lack of meaningful thresholds to be adopted, especially to monitor changes over time. Finally, based on patient’s preferences, muscle mass and strength seem to be of secondary importance compared to more comprehensive measures of functioning [48].

Regarding PROMs, currently, two different PROMs specific to sarcopenia are available: the Age-Related Muscle Loss questionnaire (ARMLQ), a PROM measuring the functional impact of reduced muscle strength from the patient perspective [49] and the SarQoL^®^ questionnaire [50, 51], a health-related quality of life questionnaire for sarcopenia. So far, only the SarQoL^®^ questionnaire has been validated with regard to the responsiveness. Geerinck et al. [40] reported that this specific instrument is more sensitive to change as compared with generic tools largely used as PROMs, such as the SF-36 or EQ-5D questionnaire. Publications [52] related to the SarQoL^®^ questionnaire also provide its standard error of measurement (2.65 points on a scale of 0–100 points) and its smallest detectable change (7.35 points on a scale of 0–100 points), through the combination of nine cohort studies, which provides a high external validity and useful data for clinical trials. Finally, this tool is available in more than 27 languages, all translations being performed by following rigorous guidelines. Because the specific questionnaires are more precise and responsive to change compared with generic questionnaires, we recommend the use of those specific instruments in clinical trials on sarcopenia. To be able to obtain a comparison with other trials and a certain generalizability of data, it is possible to combine a generic tool with a specific tool and therefore, obtain a more accurate proxy of treatment efficacy. A third PROM is currently being validated for use in sarcopenia and may provide an interesting option in the future. The Patient-Reported Outcomes Measurement Information System (PROMIS^®^) is a list of self-report measures covering multiple domains within physical, mental and social health. They have been developed as item banks, allowing for computerized adaptive testing, as well as the extraction of short form questionnaires [53]. Currently, a project funded by the Food and Drug Administration (1U01FD006887-01) is underway to certify the PROMIS measure of physical function as a clinical outcome assessment, and to investigate the specific context of use in which it could serve as a primary metric [54].

#### Secondary endpoint

Secondary endpoints in phase III studies are also necessary to determine the efficacy of a treatment and their results are expected to be consistent with the primary endpoints. Secondary outcomes are generally not considered in sample size calculation. Secondary variables are either supportive measurements related to the primary objective or measurements of effects related to the secondary objectives. Their pre-definition in the protocol is also important, as well as an explanation of their relative importance and roles in interpretation of trial results. Therefore, the statistical power to measure them is not necessarily ensured and results of secondary endpoints should be interpreted with caution. Even if, because of the reasons presented above, muscle strength and muscle mass cannot be used as primary endpoints, they are appropriate candidates for secondary endpoints. However, the limitations mentioned above regarding muscle strength and muscle mass still apply. Investigators need therefore to be cautious when using these measurements. For muscle strength, we recommend using the handgrip strength measure, because it is a highly feasible measure [55] that is part of the diagnosis of sarcopenia. The protocol of Roberts et al. [56] should be applied. Because different brands of devices could lead to different measures of strength [57], we also recommend the use of the hydraulic handheld JAMAR device (or similar) to be as standardized as possible. Unfortunately, so far, data on sensitivity of change in grip strength to interventions are still rather limited and inconsistent [58, 59]. Only one study proposed a minimal change of 6 kg (13.2 lb) in older women to be considered as clinically significant [60]. However, several non-pharmacological intervention studies have shown an increase of muscle strength following resistance exercises combined or not with nutritional interventions, which highlight the potential sensitivity to change of this measure following an intervention [14, 61]. With regard to muscle mass we recommend the use of Dual Energy X-ray absorptiometry as the current measure of choice for the assessment of fat-free mass and appendicular lean body mass, despite its well-known limited accuracy in the estimation of muscle mass and the expensive equipment. This measure has been widely used and validated reference ranges are available in the field of sarcopenia [2, 62]. CT-scans and MRI also constitute appropriate devices for the accurate measurement of muscle mass. Both tools are expensive and require certified personnel. There are other techniques, such as bioelectrical impedance analysis (BIA), ultrasound and anthropometric measures, but we presently discourage their use in a clinical trial as secondary endpoints because of their limited accuracy or insufficient published data on their validity [63–66]. At this moment more studies are needed before the relevance of D3-creatine for the assessment of muscle mass can be estimated on a sound basis. All possible primary endpoints proposed in phase II (Table 2) could also be proposed as secondary endpoints in phase III trials. This includes, as exploratory endpoints, markers of muscle quality, biomarkers of muscle metabolism and of muscle-bone interactions to support the mode of action of new chemical entities.

## Study design for pharmacological treatment RCTs in sarcopenia

The study design that should be privileged is the randomized, placebo-controlled, parallel arm trial performed in a double-blind manner. Randomization procedures should be carefully performed with stratification when needed. Primary analyses should ideally be run on an intent-to-treat basis and should include all randomized participants who received at least one dose of treatment.

The intervention should generally be a single intervention. Multi-domain interventional trials, combining several approaches in one group compared with alternative combined interventions or a control group are not recommended because it may increase the difficulty to identify the intervention which is the most effective.

A complete protocol should be registered in a trial registry before participant recruitment starts. We also encourage online publication of open-access datasets for transparency and to encourage the future realisation of individual patient data (IPD) meta-analyses.

### Length of treatment, follow-up and time point assessment

#### Phase II

Taking into account the annual rate of decline in physical performance, muscle mass and strength [67, 68], a recommendation of a minimum 6-month treatment and follow-up period was suggested upon for phase II trials. A shorter period might be acceptable depending on the mode of action.

#### Phase III

The members of the Working Group, who have a close interaction with the European regulatory authorities, feel that a minimum of 1 year of follow-up is necessary for safety issues. However, treatment studies with a longer duration are often confronted with higher numbers of dropouts which is especially relevant for older study populations. Based on this and to appreciate the offset of action, phase III clinical trial for sarcopenia may consist of a 1-year treatment period. Assessment of co-primary endpoints could be performed every 3 months. We encourage trials to use, as much as possible, similar time points for assessment to allow comparison between studies and to reduce the risk of introducing heterogeneity due to time-point assessment in meta-analyses and network meta-analyses.

### Comparator

The comparator should be placebo along with standard of care.

Because exercises and/or nutrition could be defined as usual care, these aspects should be recorded and controlled and standardized (e.g., cross countries harmonization for multicentre studies) as much as possible and should be clearly defined in the protocol. This possibility of adding standard care in the protocol of intervention is offered, considering the length of the clinical trial and to be in accordance with the WHO recommendations for encouraging of physical activity in older people. Older adults could benefit from physical activity and nutrition, both interventions having been proven for the improvement of muscle strength and physical performance [14, 61].

### Nutrition/exercise/polypharmacy record

Randomized controlled trials, despite being the best design for both Phase II and Phase III trials, would have to address numerous cofounders. Important exogenous confounders that formally require consideration are nutritional status, physical activity level, dietary pattern, comorbidities and co-prescriptions. If possible, these confounding factors should be equally matched across treatment arms. Since despite randomisation, baseline characteristics may differ between the intervention and the control group, appropriate adjustments for these differences need to be considered in the main analyses. Some subgroups might be more sensitive to the intervention than the general population. It is the responsibility of the applicant to take this possibility into account.

Ageing is associated with anabolic resistance [69]. Factors such as protein intake, vitamin D/calcium, and the acid–base balance of the diet, play an important role in maintaining muscle mass and, muscle strength and physical performance [61, 70–75]. Poor nutritional status has therefore been recognised as one of the etiologic mechanisms contributing to sarcopenia [30]. Moreover, malnutrition is a major cause of adverse health consequences, such as impaired physical function, hospitalization, and mortality in older people and could therefore impact negatively the results of clinical trials on sarcopenia [29, 76]. For these reasons, at baseline, nutritional status should be assessed and, as described in the exclusion criteria, severely malnourished participants should not be included in sarcopenia clinical trials. Besides, it is necessary to re-assess nutritional status over time during the trial, at least on each time point assessments. In addition, monitoring dietary intake during the trial is advised.

The positive impact of physical activity on muscle health is well recognized. Numerous studies have highlighted improvement of muscle mass, muscle strength and physical performance following exercises [14, 61, 77–80]. The level of physical activity within the included population is therefore an important confounding factor. We recommend recording physical activity using technological devices that may record all day activity, and not only exercises (e.g., by an inertial measurement unit, pedometers, connected watches, etc.), and, if not logistically possible, to document carefully the level of physical activity, at least on each time point assessment, through physical activity questionnaires that have been validated for use in older populations (e.g., Minnesota scale [81], IPAQ-E questionnaire, PASE [82], etc.).

The population of interest (e.g., older people suffering from muscle impairments) is a population at high risk of polypharmacy. For good clinical practice, poly-medication also needs to be carefully recorded at baseline and at each time point assessment, specifically in multi-morbid older patients with polypharmacy [83].

Finally, apart from recording major comorbidities at study entry, a measure of the global burden of comorbidities [e.g., Charlson comorbidity index or Cumulative Illness Rating Scale (CIRS-G)] may also be proposed. Thymic disorders, cognitive disorders and frailty status are also recommended to be recorded and considered as potential confounding factors.

Health events should also be recorder throughout all the study period. Among them, hospitalisations, falls, fractures, functional decline and institutionalization should be documented in each time point assessment. These health events should be considered as either as a marker of efficacy (i.e., their absence) either as an important confounder (e.g., long stay at the hospital for an infectious disease requiring a 6-week antibiotics treatment is supposed to have serious muscle consequences).

### Stratification

Stratification in clinical trials consists of partitioning subjects and results into subgroups differenced by factors other than the treatment given. To consider as many confounders or discrepant levels of response in some subgroups, performing stratification could therefore be a solution. Any subgroup analysis in phase III trials should be pre-specified. It could be therefore possible to stratify results on different parameters: on the value of primary outcome at baseline (time recorded for the 400 m walk test), on gender, on age, on comorbidities, on frailty status at baseline and, finally, on the baseline value of the variable that is intervened on (e.g., with a intervention on protein metabolism, it is possible to stratify on baseline protein intake), etc.

### Rescue medications

Given that no drugs are currently available, the issue of rescue medications is not applicable.

## Consensus

The summary of the consensus is presented in Table 3 which includes all the recommended criteria for the conduct of any new phase II and phase III pharmacological trial in sarcopenia.

**Table 3:** Recommended criteria for the conduct of any new phase II or phase III pharmacological trial in sarcopenia

	Phase II trials	Phase III trials
Appropriate study design	RCT, placebo-controlled double blind	RCT, placebo-controlled double blind
Inclusion criteria	Age 70 and +	Age 70 and +
	Able to walk	Able to walk
	Low muscle strength + low physical performance	Low muscle strength + low physical performance
Exclusion criteria	Severe malnutrition	Severe malnutrition
	Acute immobility	Acute immobility
	Specific advanced pathologies	Specific advanced pathologies
	Physical limitation attributable to a specific disease other than sarcopenia	Physical limitation attributable to a specific disease other than sarcopenia
Primary outcome	See list on Table 2	Co-primary endpoint: 1/incidence of inability to walk the 400-m walk test + 2/PROM
Secondary outcome	See list on Table 2	Muscle strength (JAMAR dynamometer)
		Muscle mass (DXA)
Length of treatment/follow-up	6 months of treatment and follow-up	1 year of treatment and follow-up
Time point assessment	Every 3 months	Every 3 months but at least every 6 months
Comparator	Placebo	Placebo
Co-treatment	Standard care for both groups	Standard care for both groups
Confounding	Nutritional status	Nutritional status
	Physical activity	Physical activity
	Co-prescriptions	Co-prescriptions
	Comorbidities	Comorbidities
	Health events	Health events
Stratification	Value of primary outcome at baseline	Value of primary outcome at baseline
	Gender	Gender
	Age	Age
	Comorbidities	Comorbidities
	Frailty status	Frailty status
	Variable intervened	Variable intervened
Rescue medication	NA	NA

## Conclusion

The present ESCEO working group was born out of concern that we may be overlooking a drug treatment for sarcopenia because we did not design the appropriate trial for this purpose. Much knowledge has been gathered in the last few years with regards to clinical trials investigating pharmaceutical interventions in sarcopenia, and the working group convened to discuss the lessons learned from recent clinical trials. The working group has formulated consensus recommendations on specific aspects of trial design, and in doing so hopes to contribute to an improvement of the methodological robustness and comparability of clinical trials, but also acknowledges that uncertainties remain. Sarcopenia remains an important challenge for ageing individuals, and it is therefore important to devote time and effort to the search for ways to prevent, slow down or treat muscle weakness.

## Appendix

See Table 4.

**Table 4:** Study protocols registered in the clinicaltrials.gov database on 22 April 2020 with sarcopenia listed as a target condition

NCT number	Status	Conditions	Interventions	Start date	Completion date
NCT04021706	Recruiting	Sarcopenia	Anamorelin hydrochloride	05-12-19	01-11-21
		Osteopenia			
NCT03995251	Recruiting	Liver cirrhosis	Testosterone	04-07-19	30-06-20
NCT03867357	Recruiting	Metastatic prostate cancer	GnRH agonist	07-12-18	31-08-21
		Androgen deprivation therapy			
NCT03788252	Active, not recruiting	Chronic kidney diseases	Renamezin	23-11-18	01-12-20
NCT03633279	Recruiting	Liver cirrhoses	Branched chain amino acid	22-06-18	01-07-20
NCT03452488	Recruiting	Sarcopenia	BIO101	24-05-18	01-12-20
		Gait disorders in old age			
		Muscle weakness			
NCT03248271	Withdrawn	Diabetes	Insulin Lispro	01-10-17	01-10-19
		Sarcopenia			
		Hypotension, orthostatic			
NCT03119610	Active, not recruiting	Obesity	Oxytocin	22-09-17	01-12-19
		Sarcopenic obesity			
		Sarcopenia			
		Aging			
		Sedentary Lifestyle			
NCT02938923	Recruiting	Hip fracture	Testosterone	15-09-17	31-05-22
		Frailty			
		Sarcopenia			
NCT03054168	Active, not recruiting	Sarcopenia	Sustanon	15-12-16	15-02-19
		Muscle hypotrophy			
		Muscle atrophy			
NCT01417364	Withdrawn	Sarcopenia	Testosterone enanthate	01-01-16	01-12-17
NCT02575235	Unknown status	Sarcopenia	CPC	01-10-15	01-08-16
NCT02594579	Unknown status	Vitamin D deficiency	Vitamin D3	01-10-15	01-12-16
		Sarcopenia			
		Critical illness			
NCT02468674	Completed	Sarcopenia	Bimagrumab	22-07-15	03-12-18
NCT01550107	Completed	Sarcopenia	Allopurinol	01-02-15	20-09-17
NCT02333331	Completed	Sarcopenia	Bimagrumab	09-12-14	28-06-18
NCT02297997	Unknown status	Sarcopenia	Cetylpyridinium chloride	01-11-14	01-11-15
NCT02370745	Completed	Sarcopenia	GLP-1	01-11-14	01-03-18
			Insulin actrapid		
			GIP		
NCT02606279	Terminated	HIV	Valsartan	01-07-14	04-08-16
		Aging			
		Sarcopenia			
		Angiotensin receptor antagonists			
NCT04354896	Completed	Subclinical hypothyroidism	Levothyroxine	01-05-14	04-05-18
		Sarcopenia			
NCT01804049	Completed	Prediabetes	Metformin	08-04-14	31-08-18
NCT03784495	Completed	Sarcopenia	Melatonin	01-01-14	01-06-15
NCT01963598	Completed	Sarcopenia	REGN1033	01-11-13	01-02-15
NCT01898611	Completed	Frailty syndrome	Ghrelin	01-07-13	31-08-16
NCT01989793	Completed	Sarcopenia	Losartan	01-07-13	01-10-16
NCT01886196	Completed	Sarcopenia	Ibuprofen	01-04-13	01-07-14
		Osteoporosis			
NCT02327091	Completed	Muscle weakness	Alfacalcidol	01-04-12	01-12-12
NCT01666522	Completed	Sarcopenia	Vitamin D	01-04-11	01-09-11
NCT02305069	Completed	Obesity	Pioglitazone	01-10-09	01-09-12
		Sarcopenia	Insulin		
			Octreotide		
NCT00891696	Completed	Sarcopenia	Rapamycin	01-04-09	01-03-15
			Sodium nitroprusside		
NCT00957801	Completed	Sarcopenia	Testosterone	01-03-09	01-12-15
			Medrol		
NCT00529659	Completed	Sarcopenia	MK-0773	01-10-07	01-10-09
NCT00509405	Completed	Healthy	Potassium citrate	01-07-07	01-05-10
NCT00475501	Completed	Male hypogonadism	Testosterone enanthate	01-01-07	01-10-14
		Muscle atrophy	Finasteride		
		Sarcopenia			
		Benign prostate hypertrophy			
NCT00315146	Completed	Obesity	Pioglitazone	01-04-06	01-04-07
		Overweight with indications for weight loss			
NCT00690534	Completed	Sarcopenia	Insulin regular	01-09-05	01-08-12
			L-NMMA		
			Sodium nitroprusside		
NCT00128115	Terminated	Hip Fracture	MK0677	01-09-05	01-08-07
NCT00240981	Terminated	Sarcopenia	Testosterone	01-01-05	01-12-09
		Hypogonadism			
		Muscular diseases			
NCT00190060	Completed	Frailty	Testosterone	01-10-04	31-12-08
		Sarcopenia			
NCT00104572	Completed	Hypogonadism	Androgel (Testosterone Gel)	01-03-04	01-01-15
		Diabetes	Anastrozole (aromatase inhibitor)		
		Sarcopenia			
		Sarcopenia			
		Depression			
NCT00183040	Completed	Sarcopenia	Testosterone	01-09-02	01-02-07
		Muscle weakness	Recombinant human growth hormone		
		Frailty			
NCT00205686	Completed	Healthy volunteers	DHEA	01-04-01	01-09-05
NCT00474279	Completed	Aging	MK-677	01-07-98	01-06-04
NCT00254371	Completed	Aging	Androgen replacement therapy	01-07-98	01-02-07
		Low DHEA for women			
		Low testosterone and DHEA for men			

Manual selection of interventional trials studying pharmacological products
